# Biased Beliefs About White Releasees’ Sensitivity to Social Pain

**DOI:** 10.1177/01461672231207952

**Published:** 2023-11-16

**Authors:** Samantha R. Pejic, Jason C. Deska

**Affiliations:** 1 Toronto Metropolitan University, Ontario, Canada

**Keywords:** social pain, pain perception, prisoners, discrimination, releases

## Abstract

The accurate perception of others’ pain is a prerequisite to provide needed support. However, social pain perception is prone to biases. Multiple characteristics of individuals bias both physical and social pain judgments (e.g., ethnicity and facial structure). The current work extends this research to a chronically stigmatized population: released prisoners (i.e., releasees). Recognizing the large United States releasee rates and the significant role support plays in successful re-integration, we conducted four studies testing whether people have biased judgments of White male releasees’ sensitivity to social pain. Compared with the noncriminally involved, people judged releasees as less sensitive to social pain in otherwise identical situations (Studies 1a–3), an effect that was mediated by perceived life hardship (Study 2). Finally, judging releasees’ as relatively insensitive to social pain undermined perceivers’ social support judgments (Study 3). The downstream consequences of these findings on re-integration success are discussed.

Prisoners and releasees comprise a large proportion of society worldwide, most predominantly in the United States, where approximately 20% of the world’s prison population is incarcerated (Walmsley, 2009; Walmsley, 2018), and over 650,000 ex-offenders are released every year (United States Department of Health and Human Services, 2022). Those involved in the criminal justice system, whether justly or unjustly, are consistently stigmatized and discriminated against both during incarceration (e.g., Clow & Leach, 2015; Clow, Ricciardelli, & Cain, 2012; MacLin & Herrera, 2006; Trammell & Chenault, 2009) and after release (e.g., Bahr et al., 2010; Pager, 2003; Roman & Travis, 2004; Travis et al., 2001; Zannella et al., 2020). Among other factors, adequate social support postrelease is vital to successful re-integration (Bahr et al., 2010). However, the provision of support is dependent on the recognition of pain (Riva et al., 2011; Summers et al., 2021), as inferences about distress directly inform a variety of prosocial responses (Kunstman & Plant, 2008; Latané & Darley, 1970). Problematically, research demonstrates that stereotypes about the resultant toughening effects of life hardship can result in decreased beliefs about how harmful socially painful events can be (Deska, Kunstman, Lloyd, et al., 2020). Relatedly, individuals deemed as having endured greater life hardship and, as a result, as being tougher, have also been found to systematically have their pain underrecognized and undertreated (Lee et al., 2019; Mende-Siedlecki et al., 2021; Trawalter et al., 2012). Although there is research examining the stigma of incarceration (for a review, see Feingold, 2021), this literature has not yet been informed by recent advances in understanding the psychological processes by which stigmatized groups are denied pain. By connecting research on prisoner stigmatization and research on pain sensitivity biases (e.g., Deska, Kunstman, Bernstein, et al., 2020), we aimed to better understand the stigmatization of released prisoners through this novel lens.

The current work addresses whether people have biased judgments about the ability for justice involved persons (i.e., releasees) to feel social pain and what factors might contribute to such a bias. Given the overlap between heightened toughness and endured life hardship stereotypes of prisoners and other stigmatized groups (e.g., Black individuals, lower socioeconomic status [SES] individuals; Hester & Gray, 2018; Hoffman & Trawalter, 2016; Summers et al., 2021; Wilson et al., 2017), the current work examined whether culturally endorsed beliefs related to perceived life hardship and toughness contribute to biased beliefs about released prisoners’ ability to experience social pain. Finally, we investigate whether such a bias might contribute to beliefs about releasees’ needing less social support to cope with their pain.

Following previous research, we operationalize *social pain* as the psychological distress experienced by aversive and upsetting social experiences including rejection, exclusion, and disrespect, that damage an individual’s social worth and interpersonal relationships (Deska, Kunstman, Lloyd, et al., 2020). Relatedly, we operationalize social support as the level of coping resources deemed necessary to cope with socially painful experiences (Deska, Kunstman, Lloyd, et al., 2020). To that end, we first briefly outline past research pertinent to the stigmatization of prisoners, both during and postrelease, with a particular focus on incarceration in the United States. We next review the emerging pain sensitivity bias literature, highlighting the conceptual overlaps between previously researched groups and the current population of focus.

## On Incarceration

People involved with the criminal justice system are among society’s most reviled groups (Crandall et al., 2002; Kjelsberg et al., 2007; Melvin et al., 1985), and comprise a nontrivial percentage of the world’s population. Since 2000, the world prison population has increased approximately 20%, with recent estimates suggesting that more than 10.74 million people are currently held in penal institutions (Walmsley, 2018). However, this figure likely underrepresents the true population because estimates cannot account for countries where figures are incomplete or unavailable, does not include prisoners held under authorities that are not recognized internationally, and does not include pre-trial prisoners in holding facilities. Therefore, the current world prison population is likely well over 11 million (Walmsley, 2018).

Understanding the worldwide incarceration rate is critical, as despite the overall upward trend of prison rates across the world, there are considerable differences both between and within nations. For instance, despite comprising approximately 4% of the world’s total population, the United States incarcerates nearly 20% of the world’s prisoners, with over 2.2 million people incarcerated at the time of writing (Walmsley, 2009, 2018). Indeed, the proportion of the U.S. population that is incarcerated has experienced a 500% increase over the past 40 years (Walmsley, 2018). One explanation for why the United States has experienced such a substantial increase in their incarceration rates is the country’s “getting tough on crime” policy (Pfaff, 2017; Tonry, 1996; Walmsley, 2018; Weidner & Frase, 2003). Rather than reflecting actual increasing crime rates, specific changes in sentencing policy (e.g., mandatory minimum sentences) has resulted in offenders remaining in prison for a longer duration than historical averages for equivalent crimes (Walmsley, 2018). This extension of sentence duration directly influences the annual number of incarcerated prisoners, which in turn translates to a greater proportion of individuals who will be stained with the stigma of incarceration once re-integrated into society. In the United States, over 650,000 ex-offenders are released from prison every year. Notably, approximately two-thirds of these releasees are re-arrested within the first 3 years of release (United States Department of Health and Human Services, 2022; United States Department of Justice, 2022). The high recidivism rates reflect not only the tremendous increase in the prison population over the past 30 years as individuals intended to exit the system ultimately return, but also the lack of social supports in the communities to which a large proportion of offenders return. Indeed, the lack of resources available to released prisoners has been cited as a major barrier to released prisoners’ prosocial integration into the community (National Institute of Justice, 2022). Recognizing the significant proportion of both prisoners, and released prisoners in the United States, we specifically focused on that context. Stigma is often described as the negative label assigned to either individuals or groups due to attributions of deviancy and/or other negative characterizations (Goffman, 1963). Associated with this negative label is the belief that those individuals are morally inferior, and as such, should be disregarded, discounted, and ultimately rejected from society (Goffman, 1963). The negative effects of stigmatization are particularly relevant for released prisoners because they are classified as violators of social norms. Consequently, criminals are one of the select populations whereby people generally feel it is socially acceptable to dislike and have openly prejudiced and dehumanizing attitudes toward (Crandall et al., 2002; Kelman, 1973; Vasiljevic & Viki, 2014). Extensive research posits that the presence of a criminal record conveys that a person is dangerous, risky, and untrustworthy (Hirschfield & Piquero, 2010), and further, that these associations persist after release (Campbell & Denov, 2004). Corroborating this, previous criminal offenders are systematically marginalized, either temporarily, or permanently, through restrictions on voting rights, housing, financial aid, employment, and additional components of community involvement (Pogorzelski et al., 2005).

Particularly problematic for released prisoners is the impact of their criminal record on the attainment of housing and employment postrelease, as access to both are vital to successful re-integration (Anderson et al., 1991; Bahr et al., 2010; Krienert, 2005; Petersilia, 2005). Unfortunately, research suggests that individuals with a criminal record experience both housing (Evans & Porter, 2015; Furst & Evans, 2017) and employment discrimination (Kohler, 1995). Indeed, research conducted with parole officers revealed that obtaining housing is one of the most significant challenges for formerly incarcerated individuals, over and above difficulties in obtaining employment (Petersilia, 2005). When evaluating rental applications, research suggests that landlords prioritize attributes such as eviction history, employment, credit, and criminal history (Clark, 2007; Healy, 2013). However, landlords are less willing to consider explanations regarding criminal history, compared with explanations of employment and income deficiencies (Clark, 2007). Furthermore, the experience of housing discrimination among released prisoners persists irrespective of crime type (Evans et al., 2019).

Together, the stigmatization prisoners both expect to, and likely will face postrelease (e.g., Crandall et al., 2002; Kelman, 1973; Vasiljevic & Viki, 2014; Winnick & Bodkin, 2008), in combination with regular experiences of discriminatory behavior (Evans & Porter, 2015; Furst & Evans, 2017; Kohler, 1995) negatively impacts the likelihood of successful rehabilitation and re-integration, one of the intended core purposes of incarceration (Benson, 2003). Research looking at recidivism factors highlights that, in addition to access to housing and employment, social support postrelease is vital to successful re-integration (Anderson et al., 1991; Bahr et al., 2010; Krienert, 2005; Petersilia, 2005). Therefore, identifying and reducing barriers to social support postrelease is critical for successful re-integration.

In addition to the notable postrelease issues releasees encounter, prisoners often suffer from poor treatment during incarceration. Indeed, research finds prisoners are exposed to contaminants and unhealthy living conditions (Awofeso, 2010; Brinkley-Rubinstein, 2013; De Viggiani, 2007), and are at risk for abuse from fellow inmates (Hochstetler et al., 2004; Kerbs & Jolley, 2007; Wolff et al., 2009) and guards (Buchanan, 2007; Hepburn, 1989). Furthermore, the harsh reality of prisons is prevalent in mass media (Cecil, 2015) and thus plays a significant role in shaping public perceptions of prisoners and prison life. As incarceration rates in the United States skyrocketed between the 1970s and 2000s, prison imagery in media transformed from a relatively uncommon occurrence to a very prevalent genre in television, film, books, and podcasts (Cecil, 2015; Cecil, 2020; Tatta & Hutton, 2002). Although personal knowledge trumps symbolic awareness (Pickett, 2014), individuals without personal experience often rely on the media as a primary source of information shaping their perceptions of prisons and prison life (Cecil, 2015). For many, popular depictions of prisons shown in television shows (e.g., Orange is the New Black; Kohan, 2013) and movies (e.g., The Green Mile; Darabont, 1999) highlighting the hardships of prison life and emphasizing toughness as major themes to surviving incarceration constitute the primary sources of information.

## Pain Sensitivity

Indeed, stereotypes about prisoners often center around hardship and toughness (Clow & Esses, 2007; Clow & Leach, 2015; MacLin & Herrera, 2006). Although little work directly assesses how these beliefs might contribute to stigmatization within this specific population, we can draw on related literatures and theoretical explanations. Most notably, the *toughness hypothesis* describes the process whereby beliefs about chronic adversity experienced by groups/individuals results in perceptions of heightened “toughness,” and subsequent minimization of pain (Summers et al., 2021). In support of this hypothesis, research demonstrates that people readily stereotype Black individuals as tougher and stronger than other racial and ethnic groups (Donovan, 2011; Hester & Gray, 2018; Wilson et al., 2017). Importantly, these judgments of Black individuals as having enhanced toughness are partially driven by judgments of Black individuals’ greater experiences of life hardship and to the lay belief that hardship fosters toughness (Cottrell & Neuberg, 2005; Harris-Lacewell, 2001; Hoffman & Trawalter, 2016; Payne, 2001). Common expressions such as “what doesn’t kill you makes you stronger,” and “no pain, no gain,” highlight this underlying “stress-as enhancing” cultural belief (Ben-Avi et al., 2018). In support of this explanation, past research indicates that people readily judge Black individuals as having more difficult lives than White individuals (Trawalter et al., 2012), in turn perceiving them as tougher, which contributes to biased beliefs about pain sensitivity (Hoffman et al., 2016; Hoffman & Trawalter, 2016).

Additional support for the theoretical connection between perceived toughness and pain sensitivity biases can be derived from recent research demonstrating that beliefs about toughness directly predict race-related biases in social pain judgments (Deska, Kunstman, Lloyd, et al., 2020). Specifically, Black targets were judged to experience more life hardship compared with White targets, which predicted judgments that Black targets were less sensitive to social pain. Notably, results from this research indicate that both Black and White participants demonstrate this bias. Although marginalized groups can internalize stigmatizing beliefs about their groups (e.g., David et al., 2019), work within pain perception specifically suggests that these biases are not driven by prejudice or dehumanization (Hoffman et al., 2016; Summers et al., 2021). Thus, the collective evidence suggests that social pain judgments are at least partially driven by toughness-related stereotypes (Deska, Kunstman, Lloyd, et al., 2020; Deska, Kunstman, Bernstein, et al., 2020). Notably, this “thick-skin” bias has also been documented among other marginalized groups. Specifically, research has links toughness beliefs with class-based biases in physical pain judgments (Summers et al., 2021, 2022); such that low-SES White targets were perceived to have experienced grater life hardship compared with high-SES White targets. Furthermore, these perceptions were associated with erroneous judgments about physical pain sensitivity.

Finally, denials of social pain to outgroups is consistent with infrahumanization (Leyens et al., 2007), which posits that outgroups are typically denied complex, secondary emotions reserved for ingroups. Indeed, some aspects of social pain are likely perceived as complex, secondary emotions (e.g., embarrassment). Furthermore, infrahumanizing judgments are linked with reductions in prosocial helping (Cuddy et al., 2007). Although more work is needed to directly test this link, it would be quite problematic if perceptions of toughness can actually result in infrahumanizing judgments.

Indeed, although toughness and resilience are often regarded as commendable attributes, research consistently demonstrates that inflated perceptions of toughness have implications for the recognition of both physical (Hoffman & Trawalter, 2016; Trawalter et al., 2012) and social pain (Deska, Kunstman, Lloyd, et al., 2020; Deska, Kunstman, Bernstein, et al., 2020). These biases in pain sensitivity are problematic because the recognition of pain is necessary for providing support (Riva et al., 2016; Summers et al., 2021). Thus, the seemingly innocuous belief that hardship toughens individuals has serious consequences for those consistently regarded as tougher, especially under conditions wherein adversity is believed to enhance resilience rather than be debilitating (Ben-Avi et al., 2018). Specifically, Ben-Avi and colleagues (2018) found that individuals who endorsed a “stress-as-enhancing” perspective were less likely to notice burnout in a hypothetical colleague. Furthermore, these individuals provided less social support to those in need compared with those who endorsed a “stress-as-debilitating” perspective.

Problematically, the endorsement of “stress-as-enhancing” perspective can be translated to race-based biases in pain perception resulting in discriminatory barriers to accessing support (Deska, Kunstman, Bernstein, et al., 2020) and pain management in health care contexts (Trawalter & Hoffman, 2016). Critically, the lay belief that life hardship leads to toughness is at odds with data showing that accumulative stress and experiences of adversity negatively impact both mental and physical health (Galea et al., 2008; Seery et al., 2010; Turner et al., 1995). Perhaps most importantly, despite perceptions of Black individuals as enduring greater life hardships (Trawalter et al., 2012), and thus being at greater risk for detrimental health concerns, racial and ethnic minorities are systematically undertreated for pain (Bonham, 2001; Drwecki et al., 2011; Freeman & Payne, 2000; Green et al., 2003; Smedley et al., 2003; Todd et al., 2000). Specifically, Black patients are less likely to be prescribed pain medication initially, and if prescribed, receive lower doses relative to White patients, as well as World Health Organization guidelines (Cleeland et al., 1997; Cleeland et al., 1994).

In the past decade, substantial research has focused on understanding biases in judgments of physical and social pain and the associated implications in health care and social support contexts, respectively (Deska et al., 2017; Deska, Kunstman, Lloyd, et al., 2020; Deska, Kunstman, Bernstein, et al., 2020; Dore et al., 2014; Hoffman et al., 2016; Hoffman & Trawalter, 2016; Mende-Siedlecki et al., 2021, 2019; Summers et al., 2021; Trawalter et al., 2012). Despite the growth of research examining biases in pain judgments, the literature has predominantly focused on race- and class-based biases (cf., Deska & Hugenberg, 2018; Summers et al., 2021, 2022). We sought to expand this literature to another group associated with toughness and hardship stereotypes: released prisoners.

Research investigating the stigmatization of prisoners has primarily been studied through the lens of Labeling Theory (Lemert, 1974; Scheff, 1966). Simply, this theory suggests that individuals with a criminal history are stigmatized due to the negative attributions associated with the “criminal” label. However, this theoretical explanation provides little room for the reduction of stigmatization, as criminal history is often static factor. Given the significant financial (Pew, 2009) and social costs (National Institute of Corrections, 2016) associated with high incarceration rates, it is integral to identify barriers to successful re-integration. Therefore, the current work aimed to explore facets of the “criminal” label that may be driving this stigmatization in efforts to provide feasible avenues for stigma reduction.

Given that judgments of prisoners as having endured greater hardships and consequently being toughened by those experiences’ parallel documented toughness and life hardship judgments of other disadvantaged groups (e.g., low-SES individuals and racial minorities), there is clear reason to predict that people might similarly see released prisoners as inured to social pain postrelease. Furthermore, as research has demonstrated a link between pain perception and support judgments (Ben-Avi et al., 2018; Summers et al., 2021), testing this hypothesis within the released prisoner population is critical as social support is consistently regarded as a significant factor contributing to recidivism (Bahr et al., 2010; Berg & Huebner, 2011). If releasees’ social pain is underrecognized, it follows that they will likely be perceived to require less coping resources. Understanding whether and how biases in social pain sensitivity contribute social support judgments about releasees is crucial to understand how to best develop interventions and suggest important areas for programming that can ease the societal reintegration process.

## The Current Work

We tested whether participants would make biased judgments about releasees’ sensitivity to social pain, whether life hardship and toughness are potential mechanisms underlying such a bias, and whether such a bias might have implications for how previously incarcerated individuals are perceived after their release. We hypothesized that participants would judge releasees’ as less sensitive to social pain than their noncriminally involved counterparts. Moreover, we predicted that perceptions of greater life hardship and, consequently, toughness would underlie this bias. Finally, we examined one potential consequence of seeing releasees as relatively inured to social pain: dampened social support judgments. To the extent that participants expected releasees to experience less social pain than control targets, we hypothesized that participants would also expect releasees to require less social support to cope with distress compared with control targets.

We tested theses hypotheses across four studies. In each, participants read brief vignettes about either a releasee or noncriminally involved control and judged how much pain they believed these targets would experience following a variety of socially painful events. In Studies 1a and 1b, we tested how participants judged the social pain sensitivity of releasee and control targets using two different measures of social pain (Deska et al., 2020; Riva et al., 2011).

Study 2 tested whether differential beliefs about life hardship and toughness between releasees and controls mediate perceptions of targets’ social pain sensitivity. Finally, Study 3 assessed a downstream consequence of this social pain bias by testing whether biased beliefs about releasees’ sensitivity to social pain predict the level of social support resources required to cope with distress.

Importantly, although the criminal label can be an invisible identity, the areas in which individuals with this label are most consistently stigmatized are those that require the disclosure of their identity (i.e., employment and housing applications). As attainment of employment and housing are critical for successful re-integration, the current work aimed to explore our hypotheses with criminal history visible to increase both the ecological validity of the study and the relevancy of the implications of our findings.

## Study 1a and 1b

In Studies 1a and 1b, we asked participants to indicate the extent to which they believe that releasees and noncriminally involved citizens are sensitive to social pain using two different measures of social pain. We used two measures to ensure any effect demonstrated was not dependent on a specific measure of social pain. Across both studies, we showed participants brief descriptions of targets that differed in social categorization (i.e., citizen and releasee) and asked them to assess how much pain they believed each would experience following aversive social events using procedures outlined in previous research (Deska, Kunstman, Lloyd, et al., 2020; Deska, Kunstman, Bernstein, et al., 2020; Riva et al., 2011). We hypothesized that, in both studies, participants would believe that releasees would be less sensitive to social pain than their citizen counterparts.

### Method

#### Statistical Power and Participants

We determined the sample size for Studies 1a and 1b based on the results of an a priori power analysis using G*Power (V3.1; Faul et al., 2007). To our knowledge, no previous published study has tested beliefs about released prisoners’ sensitivity to social pain. Thus, we used the effect size (*d* = 0.29) from previous unpublished research by the current authors focused on currently incarcerated prisoners’ sensitivity to social pain. This analysis suggested we collect at least 96 participants to obtain 80% power. To buffer against missing data, we conservatively targeted at least 150 participants for both studies. For all studies, participants were recruited via CloudResearch (Litman et al., 2017), and compensated US$0.75 toward their MTurk account for their participation. To ensure the independent nature of the studies, each study was run sequentially and we used CloudResearch’s exclusion function to ensure people only participated in one study in the package. In this and all subsequent studies, participants over the age of 18 years old who lived in the United States were eligible to participate, and no participants were excluded from analyses. Data for all studies were collected prior to data analysis, and all measures, manipulations, and exclusions are reported in these studies: https://osf.io/aymfn/?view_only=53705ea198134978a83e9c85d085f106

Participants in Study 1a were 150 MTurk workers (*M*_age_ = 39.84, *SD* = 11.12), with most self-identifying as White (72%) and male (51.30%). 150 MTurk workers participated in Study 1b (*M*_age_ = 38.89, *SD* = 9.98). Most self-identified as White (78%), one participant identified as nonbinary (0.7%), and there was an equal gender split between men (49.3%) and women (49.3%).

#### Materials

Across both studies, stimuli comprised two brief vignettes adapted from previous literature (Riva et al., 2016) that contained basic demographic information (e.g., age, name, ethnicity), and differed in social categorization (e.g., citizen, releasee). For example, “[Mark] is a 36-year-old White American citizen,” was displayed in the control condition. We controlled for race and gender as previous research highlights the robust effects of both variables (Deska, Kunstman, Lloyd, et al., 2020; Deska, Kunstman, Bernstein, et al., 2020; Riva et al., 2011; Robinson & Wise, 2003) on pain perception. We held age constant to avoid introducing that factor as a potential confound.

#### Procedure

In both studies, participants completed two trials in a randomized order. In each trial, participants viewed a vignette that contained basic demographic information (e.g., age and name) and differed in social categorization (i.e., citizen and releasee). We provided the following definitions of releasee (e.g., *A releasee is an individual who committed a crime, spent time in prison, and has been released from prison*) and social pain (e.g., *is the experience of pain as a result of interpersonal rejection or loss, such as rejection from a social group, bullying, or the loss of a loved one*; MacDonald & Jensen-Campbell, 2011). After reading each description, in Study 1a, participants rated the extent to which they believed the target would experience pain following 10 aversive social events (e.g., person’s family pet passes away; α = .89), on a 7-point Likert-type scale ranging from 1(*Not painful*) to 7(*Extremely painful;* Deska, Kunstman, Lloyd, et al., 2020; Deska, Kunstman, Bernstein, et al., 2020). In Study 1b, after reading each description, participants rated the extent to which they believed the target would experience pain following 10 different aversive social events (e.g., being ignored by a friend; α = .91), on a 7-point Likert-type scale ranging from 1 (*Not painful*) to 7 (*Extremely painful;* Riva et al., 2011). In both studies, after completing the social pain ratings, participants continued to the next trial. Finally, participants completed a demographic survey (e.g., age, racial identity, and gender) and were fully debriefed.

### Results

For both studies, we averaged participants’ social pain ratings separately for releasees, and citizens (control) to create composite scores for each social category membership. Of interest was the extent to which participants believed the targets from different social categories would experience different amounts of pain across the same situations. Paired samples t-tests revealed that in both Studies 1a and 1b, participants rated releasees as significantly less sensitive to social pain compared with controls. See Table 1 for a full breakdown of results from Studies 1a and 1b paired *t*-tests. Social pain ratings for releasee and control conditions were positively correlated with each other in Study 1a, *r*(148) = 0.58, *p* < .001, as well as Study 1b, *r*(148) = 0.72, *p* < .001.

**Table 1:** Paired Samples *t*-test Results Comparing Releasees and Controls Social Pain Sensitivity.

Study	*N*		*M*	*SD*	*t*	*df*	*p*	*d*	95% CI
1a	150	Control Releasee	4.92 4.40	.92 1.19	6.41	149	<.001	.52	[0.36, 0.68]
1b	150	Control Releasee	5.05 4.77	.88 1.21	4.13	149	<.001	.34	[0.15, 0.42]

*Note.* CI = confidence interval.

### Discussion

Studies 1a and 1b provide consistent evidence supporting our primary hypothesis. Across two studies using different measures of social pain, participants rated the releasee target as less sensitive to social pain compared with controls. Both measures were selected due to their prominence in the literature. Although items in both measures share central underlying themes (e.g., rejection, exclusion, and disrespect), they differ in their level of specificity. In general, items on the Riva measure are more general (e.g., *being humiliated in front of one’s fellows*), whereas items on Deska measure tap into similar constructs using more specific examples (e.g., *this person realizes after walking around all day that a pair of underwear was stuck to the back of their shirt*). Together, they provide converging evidence supporting the initial hypothesis.

## Study 2

Studies 1a and 1b indicated that participants believe releasees are less sensitive to social pain compared with their noncriminally involved counterparts. In Study 2, we investigated two potential mechanisms driving this pain bias. Specifically, we tested the extent to which two theoretically motivated potential mechanisms (i.e., life-hardship, toughness) serially mediated the social category membership-to-social pain link.

We measured perceived life hardship and toughness as possible mediators because recent work demonstrates the mechanistic effect of these variables on both physical and social pain perception biases (Deska, Kunstman, Lloyd, et al., 2020; Deska, Kunstman, Bernstein, et al., 2020; Hoffman & Trawalter, 2016; Summers et al., 2021). Stereotypes about prisoners are often centered around hardship and toughness (e.g., Clow & Esses, 2007). Furthermore, popular depictions of prisons highlight the hardships of prison life and emphasize toughness as major themes to surviving incarceration (Cecil, 2015). There may also be a grain of truth to certain aspects of these stereotyped beliefs. In Canada and the United States, prisoners are exposed to contaminants and unhealthy living conditions (Awofeso, 2010; Brinkley-Rubinstein, 2013; De Viggiani, 2007), and are at risk for abuse from fellow inmates (Hochstetler et al., 2004; Kerbs & Jolley, 2007; Wolff et al., 2009) and guards (Buchanan, 2007; Hepburn, 1989). These perceptions of prisoners as having endured hardships and therefore being tough map onto perceptions of Black individuals in the United states, who people similarly see as having been toughened by their exposure to hardship (Hoffman & Trawalter, 2016). Consequently, there is reason to predict that people’s perceptions of prisoners as having endured hardship, and thus being more tough, might meaningfully predict seeing them as inured to pain.

Consistent with Studies 1a and 1b, participants read descriptions of targets differing in social categorization (i.e., citizen and releasee) and assessed their social pain using the measure used in Study 1a (Deska et al., 2020; 10 items; α = .90), as this measure yielded the largest effect size (*d* = .523). Separately, participants completed measures of toughness and life hardship for each target. We hypothesized that participants would believe that releasees would be less sensitive to social pain than controls, and that judgments of life hardship and toughness would serially mediate the relationship between social category membership and social pain ratings.

### Method

#### Statistical Power and Participant

Using the averaged obtained effect sizes from Studies 1a and 1b (*d* = .43), an a priori power analysis suggested we collect at least 45 participants to obtain 80% power. To allow for a missing data buffer, we conservatively targeted at least 75 participants. We used CloudResearch to recruit 75 MTurk workers (*M*_age_ = 35.43, *SD* = 9.15). Most self-identified as White (73.33%) and male (70.67%).

#### Procedure

The procedure for Study 2 was identical to Study 1a with the notable exception that participants also completed measures of life hardship and toughness on each trial. We measured life hardship using a two-item scale adapted from the work of Trawalter et al. (2012). We used a two-item scale as previous work found that the original four-item measure did not reliably load onto a single factor (see Summers et al., 2021). Specifically, we focused on the subfactor Summers et al. termed Adversity as opposed to the Privilege subfactor, as adversity was believed to be better suited to the recently incarcerated population. The two items included in this scale were, “*How hard do you think this person’s life has been*?” and “*How much adversity do you think this person has overcome?*,” with ratings made on a 7-point Likert-type scale ranging from 1 (*Not at all*) to 7 (*Extremely*). Toughness was measured using a single, face-valid item asking participants to indicate how tough they believe the target is, on a 7-point Likert-type scale ranging from 1 (*Not tough*) to 7 (*Extremely tough*).

### Results

The first goal of the study was to provide an additional demonstration of the effect of social category membership on social pain judgments. Replicating the previous studies, participants believed that releasees (*M* = 4.07, *SD* = 1.23) were significantly less sensitive to social pain than the controls (*M* = 4.66, *SD* = 1.02), *t*(74) = 5.79, *p* < .001, *d* = 0.67, 95% CI [.39, .79]. Social pain ratings for releasee and control conditions were positively correlated with each other, *r*(73) = 0.71, *p* < .001.

Next, we tested whether participants’ judgments of perceived life hardship and toughness differed across target groups. The two individual items included in the life hardship scale were positively correlated with each other, *r*(73) = .76, *p* < .001, and so we used the composite life hardship scores in further analyses. First, participants believed that releasees (*M* = 5.17, *SD* = 0.97) endured significantly greater life hardship than the controls (*M* = 3.63, *SD* = 1.33), *t*(74) = −8.71, *p* < .001, *d* = −1.01, 95% CI [−1.89, −1.19]. Second, participants believed that releasees (*M* = 5.32, *SD* = 1.14) were significantly tougher than the controls (*M* = 4.37, *SD* = 1.04), *t*(74) = −5.58, *p* < .001, *d* = −.64, 95% CI [−1.29, −0.61]. Toughness and life hardship were moderately correlated with each other, *r*(73) = .37, *p* < .001.

Finally, we tested whether life hardship and toughness serially mediated the relationship between target social category membership and judged social pain sensitivity. We used 10,000 percentile bootstrapped samples to test the model (Montoya & Hayes, 2017). Results did not support the serial mediation, *B* = 0.10, *SE* = 0.08, 95% CI [−0.06, 0.28]. However, consistent with past research, we did observe an indirect effect of social category on social pain judgments through life hardship, *B* = 0.34, *SE* = 0.14, 95% CI [0.07, 0.63]. Finally, there was no indirect effect of social category on social pain judgments through toughness, *B* = 0.00, *SE* = 0.03, 95% CI [−0.05, 0.07]. Social pain was correlated with life hardship, *r*(73) = .46, *p* < .001, but not toughness, *r*(73) = .11, *p* = .347.

**Figure 1.**
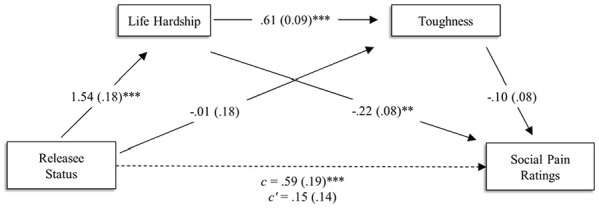
Model Showing the Effect of Releasee Status on Social Pain Judgments Through Perceived Life Hardship and Toughness in Study 2. *p < .05. **p < .01. ***p < .001.

### Discussion

Replicating the previous studies, participants in Study 2 indicated that releasees are less sensitive to social pain than control targets. We hypothesized a serial relationship such that social category membership would predict hardship judgments, which would in turn predict toughness judgments, which would in turn predict the prisoner-status bias in social pain judgments. However, this model was not supported. Instead, and consistent with past research (e.g., Deska, Kunstman, Lloyd, et al., 2020; Hoffman & Trawalter, 2016), we did observe an indirect effect of target group on social pain judgments through life hardship. One possible explanation for this finding is that the mechanistic effect of toughness on social pain ratings previously observed (Deska, Kunstman, Bernstein, et al., 2020) is driven by greater perceptions of life hardship. Previous research links life hardship to toughness (Hoffman & Trawalter, 2016), however, to our knowledge, both constructs have yet to be included in a model together. It is possible that life hardship is a stronger predictor. Given the moderate correlation between toughness and life hardship observed in the current study, combined with theoretical motivation for including both variables, this explanation seems plausible. Although these results are interesting, we urge caution in making strong inferences from them. Despite both potential mediators being theoretically motivated by existing effects in the literature, mediator tests cannot provide definitive evidence of causal relationships (e.g., Fiedler et al., 2018), plausible alternative mediators remain untested, and most effects are likely multiply determined. More research is needed to better understand the nuanced relationship between judgments of toughness, endured hardship, and social pain, and to provide a more robust understanding of the mechanism(s) underlying the previous prisoner status bias in social pain judgments. Nevertheless, the current results suggest that perceptions of life hardship may have an important role in understanding how people judge releasees’ social pain.

## Study 3

The main goal of Study 3 was to extend this research by exploring one downstream consequence of this social pain bias. We focused on the extent to which perceived social pain sensitivity in turn predicts the level of social support resources required for coping with distress. Emerging research demonstrates the biases in beliefs about who can experience pain have a range of troubling consequences (Deska, Kunstman, Lloyd, et al., 2020; Deska, Kunstman, Bernstein, et al., 2020; Hoffman & Trawalter, 2016; Trawalter et al., 2012; Waytz et al., 2015). Most germane to the current study, this research demonstrates social pain biases translate into beliefs about the level of support resources required to cope with socially painful events (Deska, Kunstman, Lloyd, et al., 2020; Summers et al., 2021).

Consistent with the previous studies, participants read descriptions of targets differing in social categorization and assessed their social pain along with the social support necessary to cope with each socially painful event. Specifically, below each social pain item was a social support item directly linked with the pain item. Participants were asked to indicate how each target should cope with the socially painful event (e.g., *How should this person cope with their best friend moving across the country?*). We hypothesized that participants would believe that the releasees are less sensitive to social pain than their noncriminally involved counterparts and, further, require fewer coping resources to deal with the same socially painful events. In addition, we hypothesized that these social pain ratings would mediate the relationship between social category membership and social support judgments.

### Method

#### Statistical Power and Participants

We targeted the same sample size for Study 3 as Study 2. Participants were 75 MTurk workers (*M*_age_ = 38.8, *SD* = 9.75) recruited via CloudResearch. Most participants self-identified as White (68%) and male (53.3%).

#### Procedure

The procedure for Study 3 was similar to Study 1a with the exception that participants also completed a social support measure on each trial. After completing social pain ratings (10 items; α = .88), we assessed social support using a 10-item measure adapted from previous research that asked participants to indicate the extent to which they believed the target should cope with each socially painful event (Deska, Kunstman, Lloyd, et al., 2020; Deska, Kunstman, Bernstein, et al., 2020). Ratings were made on a scale of 1 (*No action necessary*) to 5 (*Request formal support from a mental health professional; e.g., clinical psychologist, counselor, and psychiatrist)*, with higher scores indicating greater coping resources required (10 items; α = .88).

### Results

We turned first to the social pain measure. Replicating the previous studies, participants believed that releasee target (*M* = 4.11, *SD* = 1.00) was significantly less sensitive to social pain than the control (*M* = 4.55, *SD* = 0.79), *t*(74) = −4.47, *p* = <.001, *d* = −0.52, 95% CI [−.63, −.24]. Social pain ratings for releasee and control conditions were positively correlated with each other, *r*(73) = 0.57, *p* < .001.

Next, results on the social support measure revealed that participants believed releasees (*M* = 2.72, *SD* = .72) required significantly less coping resources than controls (*M* = 2.87, *SD* = .50), *t*(74) = −2.41, *p* = .02, *d* = −.28, 95% CI [−.27, −.03]. Social pain and support judgments were significantly correlated with each other, *r*(73) = 0.64, *p* < .001.

Finally, we tested whether social pain judgments mediated the relationship between target category and social support judgments. Using 10,000 percentile bootstrapped samples to test for a significant indirect effect, results supported this hypothesized relationship, *B* = 0.18, *SE* = 0.04, 95% CI [0.11, 0.28].

**Figure 2:**
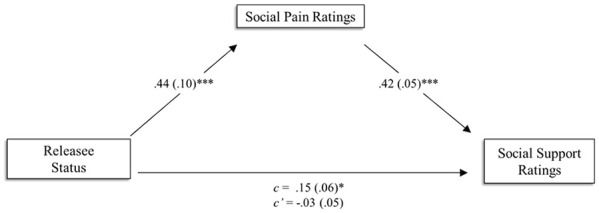
Model Showing the Effect of Releasee Status on Social Support Judgments Through Social Pain Ratings in Study 3. *p < .05. **p < .01. ***p < .001.

### Discussion

Study 3 provided further support for the primary hypothesis. Like the previous studies, participants believed that releasees are less sensitive to social pain when compared with control targets. Of additional interest was if this bias had implications for the level of coping resources required across identical socially aversive events. Results indicated that participants believe releasees required significantly fewer coping resources than control targets. Importantly, participants’ belief that releasees are less sensitive to social pain than control targets partially explains the decreased level of coping resources releasees are believed to require compared with controls. Although we do not argue that perceptions of social pain sensitivity are the sole mechanism underlying the relationship between prisoner status and social support judgments, they do appear to be important.

## General Discussion

Understanding barriers to successful re-integration is critical, especially considering that they comprise a large proportion of the U.S. population (United States Department of Health and Human Services, 2022). Importantly, adequate social support postrelease is vital to reducing recidivism (Bahr et al., 2010). However, the provision of support is dependent on the recognition of pain (Riva et al., 2011), and problematically, research suggests that individuals deemed as having endured greater life hardship and, as a result, seen as tougher, systematically have their pain underrecognized (Deska, Kunstman, Lloyd, et al., 2020). Despite the parallels between hardship and toughness judgments of released prisoners and other disadvantaged groups, the literature has yet to examine the processes by which released prisoners are denied pain. The current work sought to fill this gap in the literature by examining the disadvantages of released prisoners through the lens of biased judgments of social pain.

Studies 1a and 1b provided initial evidence that participants believe that releasees are less sensitive to social pain than control targets using two distinct measures of social pain (Deska et al., 2020; Riva et al., 2011). Study 2 had two main goals: to further demonstrate social pain biases toward releasees and to examine the potential mechanistic effects of toughness and life hardship. Results of Study 2 provided further support for this pain bias while also demonstrating the mechanistic effect of perceived life hardship. Study 3 replicated the results from the previous studies supporting this pain bias, and further demonstrated the role judgments of social pain have in beliefs about coping resources required. Importantly, these data provide further support for the negative consequences of not recognizing an individual’s pain (Deska et al., 2017; Deska, Kunstman, Lloyd, et al., 2020; Deska, Kunstman, Bernstein, et al., 2020; Hoffman et al., 2016; Hoffman & Trawalter, 2016; Mende-Siedlecki et al., 2019; Summers et al., 2021; Trawalter et al., 2012). Together, these studies consistently demonstrate that people believe released prisoners are less sensitive to social pain than nonprisoners, that this effect is at least partially driven by beliefs about how much life hardship an individual has experienced, and this bias influences beliefs about the level of social support individuals require.

### Implications

The current work integrates and builds on theory from the social perception, pain perception, and the psychology of law literatures, advancing each. These findings advance the social perception and pain perception literatures by expanding these nascent fields to an understudied, yet chronically stigmatized group: released prisoners. Notably, they provide evidence for a novel avenue through which this population might be marginalized by showing how biased impressions of releasees’ pain sensitivity might affect their post-prison treatment. Indeed, as U.S. incarceration rates continues to rise (Walmsley, 2018), so too do the number of individuals marked with stigma and dependent on adequate support resources (Berg, 2011). The prisoner stigma literature (Feingold, 2021) often conceptualizes this through the lens of stigma theory (Goffman, 1963; Herek, 2009). By focusing on biased judgments of social pain sensitivity and showcasing one underlying mechanism, the current work identifies an actionable target for future intervention. Furthermore, it suggests that focusing on pain perception biases might be an area where public policy related to rehabilitation efforts can be improved to both ease former offenders’ integration into society and reduce recidivism rates.

Despite the growing body of research focused on understanding biases in judgments of social and physical pain, this research is predominantly limited to focusing on race-based biases (Deska et al., 2017; Deska, Kunstman, Lloyd, et al., 2020; Deska, Kunstman, Bernstein, et al., 2020; Dore et al., 2014; Hoffman et al., 2016; Hoffman & Trawalter, 2016; Mende-Siedlecki et al., 2019; Trawalter et al., 2012). The current work demonstrates a social pain bias against released prisoners. Importantly, these findings have implications for identifying the potential compounded harms of underrecognized pain for those who maintain multiple stigmatized identities (e.g., Remedios & Snyder, 2018). Particularly in the United States, Black and Hispanic individuals are overrepresented in the criminal justice system (Federal Bureau of Prisons, 2021). Given that past research consistently demonstrates that Black individuals are judged relatively insensitive to physical and social pain, the current work further highlights the need for intersectional research with the pain perception literature.

### Limitations & Future Directions

The current work has several limitations that can serve as potential avenues for future research. Notably, we focused exclusively on White male targets in our vignette studies, limiting the generalizability of our findings. This design was purposeful given past research demonstrating robust effects of race (Deska, Kunstman, Lloyd, et al., 2020; Mende-Siedlecki et al., 2019) and gender (Riva et al., 2011; Robinson & Wise, 2003) on pain judgments. Indeed, the goal of the current work was to extend the social pain literature to a novel population while holding as many sociodemographic variables (e.g., race, age, gender, disability) as possible constant. Consequently, there is a great need for future work to better capture the intersectionality representative of current prison populations, especially with regards to race and gender. There are currently over 200,000 women incarcerated in the United States (The Sentencing Project, 2021), and a large majority of people involved with the criminal justice system are Black and Hispanic (Federal Bureau of Prisons, 2021). Past research indicates that people robustly judge women as more sensitive to pain than men (e.g., Deska, Kunstman, Lloyd, et al., 2020), but gender is also not perceived equivalently across race (Johnson et al., 2012). Future work should extend this nascent work on the judgments of released prisoners’ pain to women and other races and ethnicities to best understand how pain judgments create barriers to reintegration for all individuals involved in the criminal justice system.

The current studies were conducted with American participants and the vignettes depicted either “a releasee in America” or an “American citizen.” As the United States has historically taken a “get tough on crime” policy (Pfaff, 2017; Tonry, 1996; Walmsley, 2018; Weidner & Frase, 2003), as well as features large proportions of prison imagery in the media (Cecil, 2015), it is possible perceptions of prisoners in America do not accurately reflect those present in other countries (e.g., the Scandinavian prison model; Pratt & Eriksson, 2011). The United States comprises a significant portion of the total world prison population (Walmsley, 2018); however, incarceration and subsequent re-integration into the community is present all over the world. Therefore, understanding differing cultural contexts of the current effect is an important area for future research.

In addition, future research should test released prisoners’ beliefs about their own pain sensitivity. Past research focusing on race-based biases in social pain sensitivity demonstrated that both White and Black participants judge Black targets as less sensitive to pain than White targets (Deska, Kunstman, Lloyd, et al., 2020; Trawalter et al., 2012). It is unclear the extent to which released prisoners may embrace the hardness and toughness stereotypes about their social group, and whether that might affect self-pain perceptions. If so, it might have important implications for seeking needed support.

This work focuses on biased judgments about social pain sensitivity and cannot speak to accurate recognition of pain, a related but conceptually distinct question. Recent work within the context of race indicates that pain accuracy deficits stem from intergroup empathy gaps and emotion recognition deficits (e.g., Mende-Siedlecki et al., 2021; see also infrahumanization theory, Leyens et al., 2007). As important as it is to eliminate biases related to beliefs about pain sensitivity, it is equally important to reduce pain recognition accuracy deficits. Even if people believe two groups are equally *capable* of experiencing pain, they may be unwilling or unable to accurately assess others’ pain, especially if those others belong to a chronically vilified outgroup.

Finally, by identifying the existence of this social pain bias as well as at least one mechanism, the current work identifies an actionable barrier to successful re-integration. This affords the opportunity for future research to develop and test interventions designed to combat this bias and potentially reduce the discrimination individuals with a criminal history experience in accessing needed social support.
